# Association of glycosylated hemoglobin and outcomes in patients with COVID-19 and pre-existing type 2 diabetes: A protocol for systematic review and meta-analysis

**DOI:** 10.1097/MD.0000000000023392

**Published:** 2020-11-20

**Authors:** Nie Zhang, Ruiyuan Yun, Lin Liu, Liu Yang

**Affiliations:** Department of Endocrinology, Jingjiang People's Hospital, the Seventh Affiliated Hospital of Yangzhou University, Jiangsu, China.

**Keywords:** COVID-19, glycosylated hemoglobin, type 2 Diabetes

## Abstract

**Background:**

The impact of glycosylated hemoglobin on mortality in patients with coronavirus disease 2019 (COVID-19) and type 2 diabetes (T2D) remains uncertain. In this study, we aim to assess the effect of pre-hospital blood glucose regulation on patients with COVID-19 and pre-existing T2D.

**Methods:**

All randomized controlled trials (RCTs) and cohort studies of association of glycosylated hemoglobin and outcomes in patients with COVID-19 and T2D will be included in this review. PubMed, Embase, and CNKI will be searched for relevant literature, up to August 20, 2020 in English and Chinese language. Two reviewers will select trials independently for inclusion and assess trial quality. Two pairs of authors will independently extract information for each included trials. Primary outcomes are death and composite adverse outcomes: the number of participants who died or remained severely disabled. Revman 5.3 will be used for heterogeneity assessment, data synthesis, subgroup analysis, sensitivity analysisa and generating funnel-plots.

**Results:**

We will provide practical results about the association of glycosylated hemoglobin and outcomes in patients with COVID-19 and T2D.

**Conclusion:**

The stronger evidence about the association of glycosylated hemoglobin and outcomes in patients with COVID-19 and T2D will be provided for clinical practice.

**Systematic review registration:**

PROSPERO CRD42020200574.

**Ethics and dissemination:**

There is no need for ethical approval, and the review will be reported in a peer-reviewed journal.

## 1 Introduction

Type 2 Diabetes (T2D) is one of the most common metabolic disease characterized by chronic hyperglycemia. It is characterized by variable defects in both insulin secretion and insulin action.^[1]^ In the past decade, the prevalence of T2D among Chinese adults has been between 9.7% and 11.6%.^[2,3]^ T2D poses a significant economic burden and has become a severe disease worldwide.^[4,5]^

Respiratory tract infection is an infectious disease typically found in patients with T2D.^[6]^ As a respiratory tract infection, coronavirus disease 2019 (COVID-19) poses a serious threat to the health of T2D patients.^[7]^ COVID-19 is a new type of coronavirus pneumonia caused by SARS-CoV-2, which is the current challenge of global health and social issues.^[8]^ A multicenter observational study indicated that elevated fasting blood glucose within the first week of hospitalization was associated with progression to severe illness of COVID-19 in patients with pre-existing diabetes.^[9]^ Lihua Zhu et al provided clinical evidence that well-controlled blood glucose was associated with improved outcomes in infected patients.^[10]^

Glycosylated hemoglobin is an important indicator for evaluating blood sugar control in patients with T2D. A high glycosylated hemoglobin increases the hospitalization risk of patients with T2D owing to pneumonia.^[11]^ Ayman A Al Hayek et al observed that high glycosylated hemoglobin level was a significant risk factor for the hospital admission among COVID-19 patients with diabetes. Another study indicated glycosylated hemoglobin was a predictor of COVID-19 severity in patients with diabetes.^[12]^ Zhenzhou Wang found that high glycosylated hemoglobin level was associated with inflammation, hypercoagulability, and low SaO_2_ in COVID-19 patients.^[13]^ These studies suggest the correlation between glycosylated hemoglobin and the prognosis of COVID-19 patients.

In this study, we aim to conduct a meta- analysis of cohort studies to assess the association of glycosylated hemoglobin and outcomes in patients with COVID-19 and T2D, to provide stronger evidence for the clinic practice.

## 2 Methods

### 2.1 Study registration

This protocol will be performed in comply with Preferred Reporting Items for Systematic Review and Meta-Analysis Protocols (PRISMA-P) guidance^[14,15]^ and has been registered with the International Prospective Register of Systematic Reviews (PROSPERO) in August 2020 as CRD42020200574.

### 2.2 Inclusion and exclusion criteria

#### 2.2.1 Types of studies

All randomized controlled trials (RCTs) and cohort studies of association of glycosylated hemoglobin and outcomes in patients with COVID-19 and T2D will be included in this review.

#### 2.2.2 Types of participants

The diagnosis of COVID-19 was confirmed as positive result for respiratory pathogen nucleic acid test and nasopharyngeal swab with real-time reverse transcriptase polymerase chain reaction (RT-PCR) or high-throughput sequencing. T2D is due to a progressive loss of β-cell insulin secretion frequently on the background of insulin resistance, and according to the Standards of Medical Care for T2D in China 2019,^[16]^ the diagnostic criteria for diabetes are

1. typical symptoms of diabetes (polydipsia, polyuria, polyphagia, and weight loss) plus random plasma glucose ≥11.1 mmol/L or
2. fasting plasma glucose (FPG) ≥7.0 mmol/L or
3. oral glucose tolerance test (OGTT) 2-Hour plasma glucose (2hPG) ≥11.1 mmol/L.

#### 2.2.3 Types of interventions

Patients are divided into well-controlled blood sugar group (glycosylated hemoglobin ≤7%) and poorly-controlled blood sugar group (glycosylated hemoglobin >7%).

#### 2.2.4 Types of outcome assessments

Death and composite adverse outcomes: the number of participants who died or remained severely disabled.

### 2.3 Search strategy

Electronic databases including PubMed, Embase, and China National Knowledge Infrastructure (CNKI) will be searched for relevant literature, up to August 20, 2020 in the English and Chinese language. The key search terms will be used are [“Hemoglobin A, Glycosylated” OR “Hb A1c” OR “HbA1” OR “Glycosylated Hemoglobin A” OR “Hemoglobin A, Glycosylated” OR “Hb A1” OR “Glycohemoglobin A” OR “Hemoglobin A(1)” OR “Hb A1a-2” OR “Hemoglobin, Glycosylated A1a-2” OR “A1a-2 Hemoglobin, Glycosylated” OR “Glycosylated A1a-2 Hemoglobin” OR “Hemoglobin, Glycosylated A1a 2” OR “Hemoglobin, Glycosylated A1a-1” OR “A1a-1 Hemoglobin, Glycosylated” OR “Glycosylated A1a-1 Hemoglobin” OR “Hemoglobin, Glycosylated A1a 1” AND “Diabetes Mellitus, Noninsulin-Dependent“ OR “Diabetes Mellitus, Ketosis-Resistant” OR “Diabetes Mellitus, Ketosis Resistant” OR “Ketosis-Resistant Diabetes Mellitus” OR “Diabetes Mellitus, Non Insulin Dependent” OR “Diabetes Mellitus, Non-Insulin-Dependent” OR “Non-Insulin-Dependent Diabetes Mellitus” OR “Diabetes Mellitus, Stable” OR “Stable Diabetes Mellitus” OR “Diabetes Mellitus, Type II” OR “Type 2 Diabetes” OR “Diabetes, Type 2” AND “2019 novel coronavirus vaccine” OR “2019-nCoV vaccine” OR “COVID-19 virus vaccine” OR “COVID19 virus vaccine” OR “coronavirus disease-19 vaccine” OR “SARS2 vaccine” OR “coronavirus disease 2019 virus vaccine” OR “Wuhan coronavirus vaccine” OR “novel coronavirus vaccine” OR “coronavirus disease 2019 vaccine” OR “COVID19 vaccine” OR “SARS-CoV-2 vaccine” OR “BNT162 vaccine” OR “mRNA-1273 vaccine” OR “mRNA 1273 vaccine” OR “mRNA-1273 2019-nCoV vaccine” OR “COVID-19 vaccine mRNA-1273” OR “mRNA-1273 COVID-19 vaccine” OR “2019-nCoV vaccine mRNA-1273” OR “PittCoVacc” OR “microneedle arrays SARS-CoV-2 S1 subunit vaccines” OR “MNA SARS-CoV-2 S1 subunit vaccines” OR “INO-4800 vaccine” OR “Covid-19 aAPC vaccine” OR “COVID-19 artificial antigen presenting cells vaccine”]

### 2.4 Data collection

#### 2.4.1 Selection of studies

**Figure 1:**
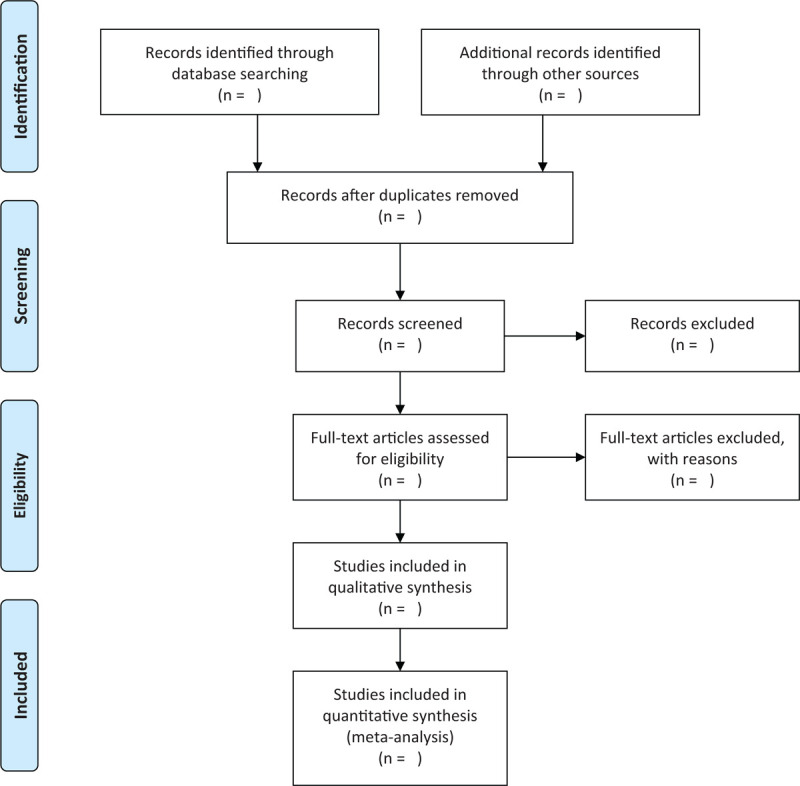
Flow diagram of the study selection process. *From:* Moher D, Liberati A, Tetzlaff J, Altman DG, The PRISMA Group (2009). *P*referred *R*eporting *I*tems for *S*ystematic Reviews and *M*eta-*A*nalyses: The PRISMA Statement. PLoS Med 6(6): e1000097. doi:10.1371/journal.pmed1000097.

A pair of reviewers will select trials for inclusion independently. The flow diagram of the study selection process is shown in Fig. 1.

#### 2.4.2 Data and information extraction

We will independently select trials for inclusion and extract the data. Two pairs of review authors will independently extract data for outcome assessments (the number of participants who died or remained severely disabled) for each included trial and whether all the participants will be accounted for in the analysis. The fifth author will check all the data. We will resolve disagreements in the numbers extracted by discussion.

### 2.5 Assessment of risk of bias

The review authors will independently assess the quality of the trials included in the review by assessing the following items:

1. Allocation concealment (selection bias);
2. Blinding (performance bias and detection bias);
3. Blinding of participants and personnel (performance bias);
4. Blinding of outcome assessment (detection bias);
5. Incomplete outcome data (attrition bias);
6. Selective reporting (reporting bias).

The fifth author will check all the data. We will use this information to evaluate quality and resolve disagreements by discussion until consensus is reached.

### 2.6 Data analysis

#### 2.6.1 Assessment of heterogeneity

The Chi-Squared test and *I*^2^ statistic will be used to assess heterogeneity. It indicates that the heterogeneity is in the acceptable range when *P* > .10 and *I*^2^ < 50%. If the heterogeneity exceeds the acceptable range (*P* < .10 or *I*^2^ > 50%), the random effect model shall be used for data analysis; otherwise, the fixed effect model will be adopted.

#### 2.6.2 Date synthesis.

Review Manager 5.3 will be used to assess the risk of bias, heterogeneity, sensitivity and subgroup analysis. We calculate a weighted estimate of the treatment effect across trials and for the interpretation of the results, and 95% CI will be used. *P* < .05 will be considered statistically significant.

#### 2.6.3 Subgroup analysis

We will emplore the subgroup analysis of randomized and non-randomised to explore the possible causes of high heterogeneity.

#### 2.6.4 Sensitivity analysis

Sensitivity analysis will be conducted by excluding trails one by one and observe whether the synthesis result changes significantly. If there are significant changes, we will make a decision cautiously to decide whether to merge it. If the changes not significantly, it indicates that our result is firm.

### 2.7 Assessment of publication bias

If there are more than 10 articles available for analysis, funnel plots will be generated to assess publication bias. A symmetrical distribution of funnel plot data indicates that there is no publication bias, otherwise, we will analyze the potential reasons for this outcome and give reasonable interpretation for asymmetric funnel plots.

### 2.8 Confidence in cumulative evidence

GRADE system will be used for assessing the quality of our evidence. According to the grading system, the level of evidence will be rated high, moderate, low and very low.

## 3 Discussion

T2D patients might develop a deregulated immune system, predisposing to various infections.^[17]^ T2D has been linked to poor outcomes of COVID-19,^[18–21]^ but the relationship between COVID-19 and pre-infection glycemic control is still unclear. Glycosylated hemoglobin is a biomarker with a central role in the diagnosis and follow-up of patients with diabetes.^[22]^ A study pointed that inflammations markers such as C reactive protein (CRP) level, serum ferritin level, and erythrocyte sedimentation rate (ESR) in COVID-19 cases were positively correlated with glycosylated hemoglobin, while SaO_2_ was negatively correlated with glycosylated hemoglobin,^[13]^ suggesting glycosylated hemoglobin test after admission is helpful for assessing inflammation, hypercoagulability and prognosis.

This study will provide the current evidence for the impact of glycosylated hemoglobin on the prognosis of COVID-19 patients with T2D and guide the individualized treatment of COVID-19 patients.

## Author contributions

**Conceptualization:** Nie Zhang, Liu Yang.

**Methodology:** Nie Zhang, Ruiyuan Yun, Lin Liu, Liu Yang.

**Writing – original draft:** Nie Zhang, Ruiyuan Yun, Lin Liu.

**Writing – review & editing:** Nie Zhang, Liu Yang.
